# Influence of Hydrofluoric and Nitric Acid Pre-Treatment and Type of Adhesive Cement on Retention of Zirconia Crowns

**DOI:** 10.3390/ma14040960

**Published:** 2021-02-18

**Authors:** Osama Harb, Walid Al-Zordk, Mutlu Özcan, Amal Abdelsamad Sakrana

**Affiliations:** 1 Fixed Prosthodontics Department, Faculty of Dentistry, Mansoura and Horus University, Mansoura 35511, Egypt; Osamaharp89@gmail.com (O.H.); walidwa@gmail.com (W.A.-Z.); 2 Center for Dental and Oral Medicine, Division of Dental Biomaterials, Clinic for Reconstructive Dentistry, University of Zurich, 8032 Zurich, Switzerland; mutluozcan@hotmail.com

**Keywords:** translucent zirconia, oxide ceramics, MDP-containing cement, adhesive cement, dental restoration

## Abstract

Background: The aim of this study was to test the impact of hot acids etching and two types of adhesive cement on the retention of zirconia crowns. Methods: Forty maxillary premolars were prepared, and zirconia crowns were designed and fabricated with proximal extensions, then divided into 4 groups (n = 10). Group AP; the crowns were air-abraded and cemented using Panavia SA Cement. Group AL; the crowns were air- abraded and cemented using GC LinkForce. Group AHP; the crowns were air-abraded, etched with the hot acids (48% hydrofluoric acid and 69% nitric acid), and cemented using Panavia SA Cement. Group AHL; the crowns were air-abraded, etched with the hot acids, and cemented using GC LinkForce. Each zirconia crown was pre-treated and bonded to its corresponding tooth. After thermocycling (5–55 °C/10,000), the retention test was performed and the load required to dislodge the crown was reported in Newton (N), and mode of failure was recorded. The retention strength (MPa) was calculated for each tested variable and statistically analyzed. Results: Group AHP showed the highest mean value of the retention strength, followed by group AP then group AHL. Group AL showed the lowest value. A statistically significant effect (*p* = 0.001) of the hot acids etching on the retention of zirconia crown was found. Also, there was a significant effect (*p* = 0.000) of the cement type. The interaction between surface treatment and the cement type has no significant impact (*p* = 0.882). The main mode of failure for Panavia SA Cement is mixed mode of failure, while for G-CEM LinkForce is adhesive failure. *Conclusions*: Hot acid etching pre-treatment improved the retention of zirconia crown. Usage of Panavia SA Cement with hot acids etching is effective can be used for adhesive cementation of zirconia crown.

## 1. Introduction

As a dental biomaterial zirconia represents an attractive restorative option in fixed prosthodontics, with many advantages such as excellent biocompatibility and good mechanical characteristics and acceptable optical properties [1,2]. However, its great surface stability creates several problems as regards the efficiency and durability of the chemical or mechanical bond with different cementation systems [3]. It is very necessary to properly cement a fixed prosthesis and it has been found that surface treatment before cementation will improve the bonding [4,5]. Owing to the lack of glassy matrix in the zirconia microstructure, the conventional mechanical and chemical strategies used with glass-ceramics are not pertinent for use with zirconia [6,7].

For a stable bond between cement and zirconia, there should be micro-mechanical retention and chemical bonding [3,8]. It was reported that advanced adhesive systems, beside different surface treatment are useful for improving bonding to zirconia ceramics [9,10]. Various surface pre-treatment methods were employed to improve bonding with zirconia, such as air abrasion using aluminum oxide particles, pyrochemical silica application and hot chemical pre-treatment [11]. Air-borne particle abrasion with aluminum oxide particles is simple and commonly used method to increase the wettability, surface roughness, surface area for micromechanical interlocking [12,13]. However, airborne particle abrasion results in structural defects and induction of sharp cracks that enhance radial cracking during function [3,14]. Zirconia are non-silicate ceramics and cannot be etched with traditional acids [15]. Therefore, another efficient conditioning can be employed for zirconia, such as piranha solution and hot acids etching [16,17,18,19,20].

Retention of the fixed restorations are influenced by variables such as the geometry of tooth preparation [21], the relative adaptation of the restoration [22], and the cement type [23,24,25]. Dental cements should form a strong bond to tooth structure and restoration, and have high strength under tensile forces [26]. The retention mechanism can be from friction, micro-mechanical or chemical bonding or combination [27]. The increasing popularity of self-adhesive resin cements is attributed to its simplify, time-saving, and adhesive properties [28,29,30,31,32]. Previous studies recorded satisfactory results with the application of MDP-containing resin cement [33,34,35,36].

Shear, tensile, microtensile or retention tests, are commonly employed to determine the bonding performance of a luting cement [37,38,39,40,41]. The retention test was employed by many researches, where the cemented restoration is subjected to axial dislodging forces till failure [39,42,43]. The purpose of this in-vitro investigation was to evaluate the influence of hot acids chemical pre-treatment and the cement type (Panavia SA cement (Kuraray Noritake Dental Inc., Okayama, Japan) plus and G-CEM LinkForce cement (GC America Inc., Chicago, IL, USA) on the retention of monolithic zirconia restoration. The null hypothesis tested was that the hot acids will enhance the retention of zirconia crown. The second null hypothesis was that the retention of cemented zirconia crown will not be affected by the cement type.

## 2. Materials and Methods

Forty maxillary first premolars extracted for orthodontic reason were collected for the current investigation. Each included tooth was free from caries, cracks, or fractures. Approval to use human teeth was obtained from the Research Ethics Committee (No.02021018) at the Faculty of Dentistry, Mansoura University, Egypt. All teeth were measured to included only that with similar dimensions (bucco-lingual and mesio-distal) [44]. Each tooth was debrided and disinfected, then kept in in standardized saline at room temperature. As shown in Table 1, all teeth were classified into 4 groups (n = 10). The root of each tooth was drilled with fissure bur at high speed to allow placement of an orthodontic wire in order to avoid any movement of tooth during the retention test. Each tooth was centrally mounted in acrylic resin block.

To standardize the teeth preparation, the CAD-CAM technology was utilized [45]. A single tooth was prepared with aid of dental surveyor (Dentalfarm A3006 B manual surveyor, Turin, Italy). The preparation has 1.5 mm centric cusp reduction and 1 mm non-centric cusp reduction, 0.5 mm chamfer margin, and 6-degree taper. Then, the tooth was scanned with an optical scanner (Ceramil Map 400 Amann Girrbach, Koblach, Austria), and the scanned data were modified using a software (Rhinoceros business 3D, Robert McNeel & Associates, Seattle, WA, USA). Then, the scanned tooth was converted to 3D designs using (3 shape 3D viewer, Tais Clausen and Nikolaj Deichmann, Copenhagen, Denmark). Thus, before moving to reproduction, the designed model were brought to completion.

Each prepared tooth was scanned (Identica Hybrid, MEDIT Corp, Seoul, Korea) and 80 μm cement space was selected. The crowns were designed with mesial and distal projections which were made to assist restoration removal after cementation during the retention test. After designing of the crowns, they were dry milled from zirconia block (DD CubeX2 zirconia, Dental Direkt GbmH, Spenge, Germany) using CAD-CAM milling machine (CORiTEC 250i touch, imes-icore GmbH, Eiterfeld, Germany). The milled crowns were sintered (Tabeo-1/M/Zirkon-100, MIHM-VOGT, Stutensee, Germany), then glazed based on the recommendations of the manufacturer.

For each prepared tooth, the STL (Standard Tessellation Language) file of each prepared tooth was manipulated through specific 3D computer graphic software (MeshLab software, Istituto di Scienzae Tecnologie dell’Informazione, Roma, Italy) to determine and calculate the surface area of each preparation.

All zirconia crowns were air-borne particle abraded (Basic classic fine sandblasting unit, Renfert, Hilzingen, Germany) with 50 μm aluminum oxide, at 2 bar for 10 s. Then, the crowns were rinsed and dried.

For groups AHP and AHL, zirconia crowns were etched using a mixture (1:1) of 48% hydrofluoric acid (HF) and 69% Nitric acid (HNO_3_) [46]. The prepared acid mixture was injected into the fitting surface of the crowns, then placed in an oven (BINDER D78532 Tuttlingen, Germany) at 100 °C for 25 min. Finally, the crowns were rinsed and dried.

Groups AHP and AP, the cementation was performed using Panavia SA (Kuraray Noritake Dental Inc., Okayama, Japan) based on the recommendations of the manufacturer. For each restoration, the cement was injected into the fitting surface, then seated over its corresponding tooth, and held under 10 N constant load. Finally, each surface was light cured for 20 s [47]. 

For groups AHP and AP, the cementation was performed using G-CEM Link Force cement (GC America Inc.). First, the bond (G-Premio bond, GC America Inc.) was applied over the prepared surface of the tooth with rubbing motion for 10 s, air dried for 5 s, and light cured for 10 s based on the recommendations of the manufacturer. Then, the primer (G-multi primer, GC America Inc.) was applied into the fitting surface of the crown and dried. Finally, the cement was injected into the fitting surface, then, the crown was seated over its corresponding tooth. The crown was held under 10 N constant load, and light cured at each surface for 20 s.

In order to mimic the intra oral conditions, the cemented specimens were subjected to thermocycling (SD Mechatronic thermocycler THE-1100, SD Me­chatronics, Westerham, Germany). The thermocycling was performed for 10,000 cycles between 5–55 °C (25 s dwell time).

**Figure 1:**
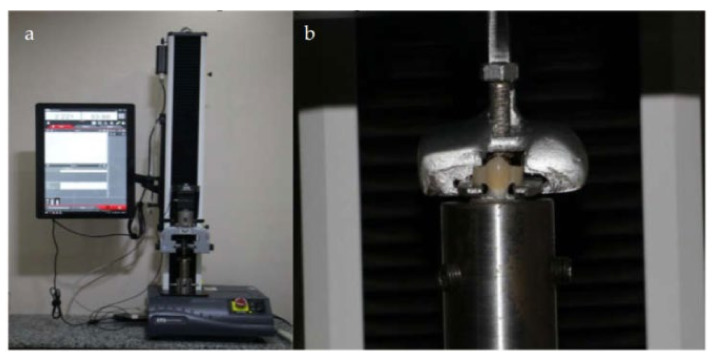
The retention test. Panel (**a**) The specimen fixed within the universal testing machine. Panel (**b**) The restoration suspended using a specific tool from the upper part of the universal testing machine.

Using a universal testing machine (Instron, 2519-104, 3345, Canton, MA, USA,) at crosshead speed of 0.5 mm/min, each crown was subjected to axial dislodgment forces until failure (Figure 1a). The specimen was attached by tightening screws to the lower fixed part of the device, and the crown was suspended from the upper moving part of the device through a specific tool fabricated to accommodate the crown projections (Figure 1b). The crown was submitted to a gradually increasing axial force (0.5 mm/min) until failure occurred; the failure load was reported in Newton (N).

After retention test, each specimen was observed by a single operator (O.H.) under ×20 magnification (Olympus SZ 61, Tokyo, Japan) to identify the failure mode. The failure mode was recorded as: adhesive (with the cement interface), cohesive (within the restoration or the tooth substrate), or mixed. Then, representative specimens were examined using Scanning Electron microscopy (JSM-6510LV, JEOl Ltd., Tokyo, Japan).

Statistical interpretations were done using the SPSS software (SPSS Statistics for Windows v25.0 SPSS Inc., Chicago, IL, USA). Regarding the retention strength, the Shapiro-Wilk test displayed that the data revealed normal distribution (df = 10, *P* > 5% for each group). To evaluate the interaction of the 2 independent variables (hot acids etching and cement type, regarding the retention strength), two-way ANOVA (Analysis of Variance) test was performed.

## 3. Results

Group AHP showed the highest mean value of the retention strength, followed group AP, group AHL and Group AL revealed the lowest mean value of the retention strength (Table 2).

Regarding the retention strength of zirconia crown, there was significant difference (*p* < 0.05) between study groups, as indicated by Post Hoc test, with the highest mean retention strength value within group AHP (Table 3). Also, a significant difference (*p* = 0.000) was revealed between group AHL and group AP with the higher retention strength with group AP. Additionally, a significant difference (*p* = 0.014) was shown between group AHL and group AL with the higher retention strength with group AHL. Finally, there was significant difference between (*p* = 0.000) group AP and group AL with the higher retention strength with group AP.

Two-way ANOVA test showed a significant effect (*p* = 0.001) from using the hot acid pre-treatment on the mean value of the retention strength. Also, a significant effect (*p* = 0.000) was revealed regarding the cement type. The interaction between surface treatment and the type of resin cement has no significant effect (*p* = 0.882), as shown in Table 4.

**Figure 2:**
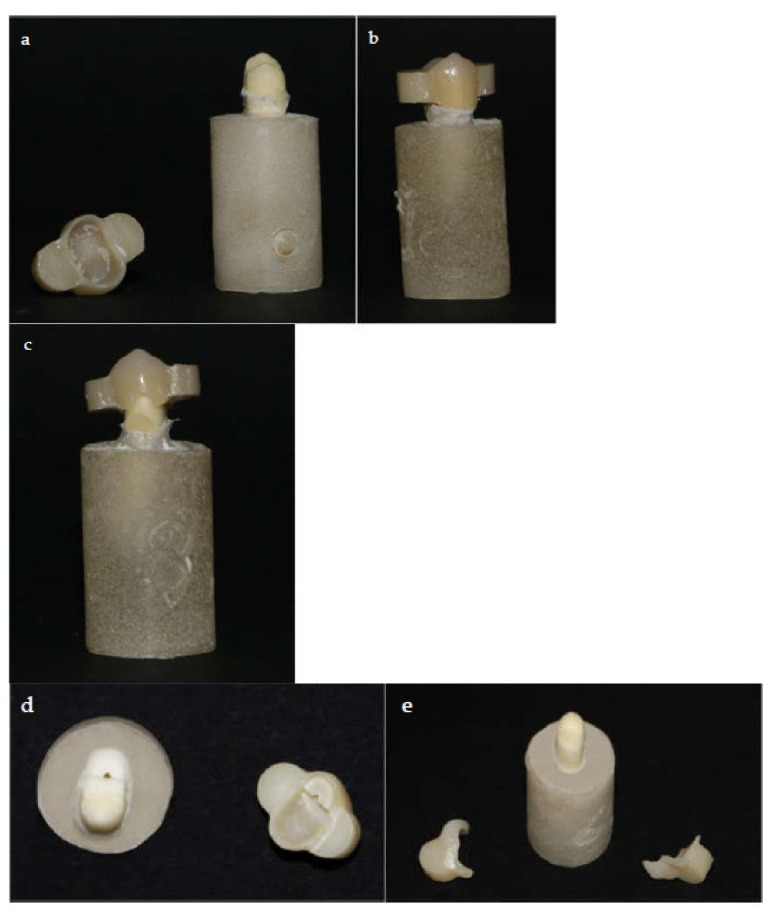
The mode of failure within study groups. Panel (**a**) adhesive failure group AL. Panel (**b**) Cohesive failure group AHP. Panel (**c**) Mixed failure group AHL. Panel (**d**) Mixed failure group AHP. Panel (**e**) Mixed failure group AP.

The modes of failure of all tested specimens are presented in Table 5 and Figure 2. The main mode of failure for Panavia SA Cement Plus is mixed mode of failure, while for G-CEM LinkForce Cement it is adhesive failure. Using of hot acids after air-borne particle abrasion decreased the adhesive failure with G-CEM LinkForce Cement.

**Figure 3:**
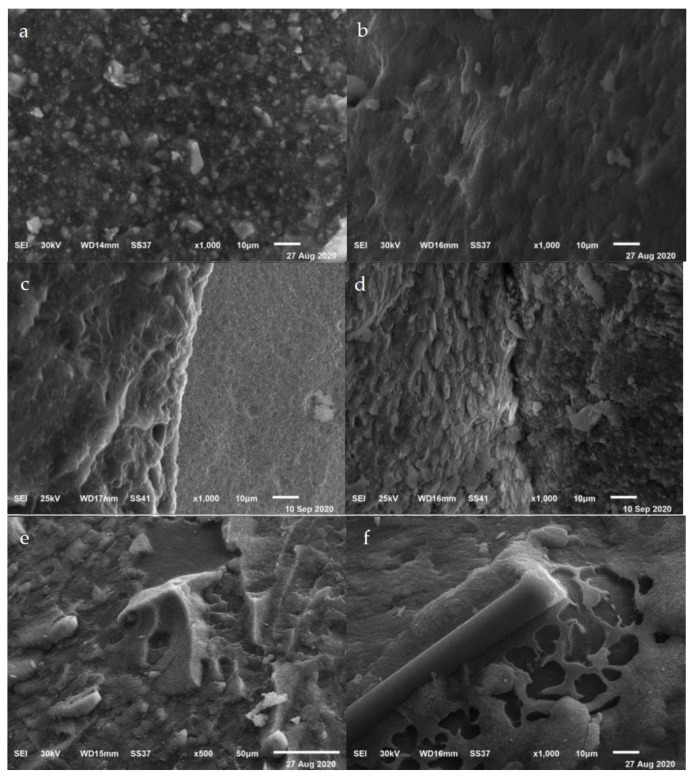
SEM micrographs. Panel (**a**) Rough surface with scattered remaining cement on the zirconia surface from group AP. Panel (**b**) Wide distribution of a porous zirconia surface with scattered remaining cement from group AHL. Panel (**c**) Adhesive failure within the cement layer from group AHL. Panel (**d**) Mixed failure within tooth and cement material from group AP. Panel (**e**) Cement remnants on tooth after adhesive failure from group AHL. Panel (**f**) Cement remnants on tooth after adhesive failure from group AHL.

Figure 3 shows scanning electron microscope images of specimens after the retention test. Rough surfaces with scattered remaining cement were detected on the zirconia crowns in group AP, as shown in Figure 3a. Figure 3b reveals a wide distribution of pores on the surface of zirconia crowns with scattered remaining cement in group AHL. Moreover, adhesive failure occurred in the cement material in group AHL, as observed in Figure 3c. Figure 3d shows mixed failure of both the tooth and cement material. However, using hot acids after air-borne particle abrasion increased the surface roughness a with wide distribution of a network of pores of different depth and width as in group AHL (Figure 3e,f). Scattered cement remaining on the tooth surface after adhesive failure was observed in group AHL (Figure 3e,f).

## 4. Discussion

The proposed first null hypothesis in the current investigation was that hot acids would enhance the retention of zirconia crowns. The second null hypothesis was that the type of adhesive resin cement would not affect the retention of zirconia crowns. The results of the current investigation partially support the null hypotheses, as the hot acid etching improved the retention of the zirconia restoration crowns. The type of the adhesive resin cement, however, also influenced the retention of the zirconia crowns.

Human teeth were utilized in the current study because of their bonding features, thermal characteristics, modulus of elasticity and strength that are closer to the clinical circumstances [44]. To prepare the teeth, the CAD-CAM technology has been used since it provides standard preparation of all teeth for in vitro tests and provides a reproducible tool that can be used in experiments to facilitate comparisons and minimize bias arising from differences in the processing of samples [45].

Clinically, the cemented dental restorations are exposed to adverse conditions, and artificial aging can be performed to mimic the intra-oral circumstances [48]. In the present study, thermal aging was performed to mimic oral conditions that were proved to have an effect on a retention of dental restorations.

In this in vitro investigation, retention tests were used because they have many benefits such as easy conduction, good reproducibility as well as the percentage of bonded to non-bonded areas in restorations cemented on abutments or dies is far higher than that seen in ceramic or composite cylinders cemented on flat surfaces [48]. Many precautions were taken to ensure accurate results such as standard preparation of teeth and accurate measurement of surface areas [48]. In the present study, restoration was designed to have mesial and distal projections to assist its removal during the retention test [38]. Factors such as standardized tooth reduction and exclusion of the shear stresses during testing, had to be managed with retention test [39]. Shear bond strength is a popular test, however it may result in cohesive failure due to inhomogeneous stress distribution at the bonding interference area [37]. Regarding tensile strength tests, the difficulty of sample alignment also may result in uneven stress distributions [10].

The zirconia restorations were treated with air abrasion using 50 μm alumina particles in the current investigation. This is among the most commonly used methods for surface pre-treatment that can be easily performed with excellent results in increasing surface roughness, micromechanical undercuts and improve bonding of zirconia [8,25,40,49]. It was reported that air-borne particle abrasion improves the crown retention regardless of the type of luting material [49]. Air-borne particle abrasion of size 50 μm had less harmful effects on zirconia when compared to air-borne particle abrasion of 120 μm size at the same pressure [5]. However, owing to the formation of surface micro-cracks, it could have a harmful impact on the zirconia surface, which may decrease the flexural strength [11,14]. The impact of air abrasion on the flexural strength of zirconia is controversial in terms of duration and power of the procedure [5,20].

The hot acid etching used in this study was prepared according to Liu et al [46] who confirmed that etching of the zirconia using a mixture of HF acid of concentration 48% and HNO_3_ of concentration 69% with a 1:1 proportion at 100 °C for 25 min, leads to an improved dissolution rate of zirconia grains, surface roughness and bonding surface area to cement, with no phase transformation.

The results of the current study have indicated a significant effect of the surface treatments used along with the cements used. Group AHP showed the highest mean value of retention strength (3.83 ± 0.71 MPa), followed by group AP (3.22 ± 0.35 MPa) and group AHL (2.00 ± 0.52 MPa). Group AL showed the lowest mean value of retention strength (1.44 ± 0.22 MPa). The hot acid etching after air-borne particle abrasion improved the retention significantly. Liu et al. [46] observed higher bonding values of zirconia treated with hot acid etching to resin cements over other surface treatments. The corrosive activity of the HF solution as well as the rate of dissolution could be enhanced by heating to an elevated temperature. Another study found pre-treatment of zirconia with hot acid mixtures (69% nitric acid and 48% hydrofluoric acid) after air-borne particles abrasion enhanced the bond between zirconia and the resin cement, and the specimens revealed a wider distribution roughness which result in micromechanical retention and better bonding strength [35]. Furthermore, Monteiro et al. [43] evaluated the impact of various pre-treatment methods after aging and concluded that, relative to air-borne particle abrasion, tribochemical silica coating did not offer extra retention for zirconia restorations.

In the present study, Panavia SA Cement Plus gave better retention with zirconia crowns. This may be explained by its good hydrolytic stability related to functional monomer that have long carbonyl chain [13]. G-CEM LinkForce Cement is a self-etch system requiring at least two steps; primer application then cement application, which might increase the window of contamination and decrease bond strength [36]. On the other hand, Higashi et al. [30] reported that self-adhesive resin cement has inferior bond strength with zirconia, however their results could be attributed to the hand mixing of Panavia SA Cement which may led to formation of bubbles in the cement mix and very likely played a role in decreasing the mechanical properties of this resin cements. Additionally, another study concluded that the retention of the zirconia coping is not affected by the cement type and only good preparation geometry of the preparation enhanced its retentive quality [41].

In the current research, mixed failure was the principle mode of failure when using Panavia SA Cement Plus. Adhesive failure, which can be attributed to poor bonding between the zirconia and cement due to cement deterioration from thermocycling was the cause of failure of G-CEM LinkForce Cement [50]. Elhers et al. [48] assessed the retentive strength values of self-adhesives and self-etch cements and concluded that the retentive strength values after thermal aging varies greatly between cements.

Regarding the scanning electron microscope evaluation, the zirconia surface appeared rough with irregular grooves after using air-borne particle abrasion [38,49]. However, with hot acid etching after air-borne particle abrasion, the roughness increased with wide distribution of pores of different depth and width in the network. This finding is consistent with Liu et al. [46], who found that the use of hot acid etching induced the dissolution of zirconia grains and resulted in the formation of several craters of different sizes and thus improved the roughness of the surface of zirconia relative to air-borne abrasion.

As a limitation for this in vitro study, the phase transformation and mechanical properties of zirconia crowns was not evaluated after applying the surface pre-treatment. Also, additional types of resin cements need to be investigated. Additionally, further clinical trials will certainly be needed to corroborate these in vitro results. Finally, the hot acid etching need to be evaluated against more methods employed as a zirconia treatment method.

## 5. Conclusions

Based on the results of this in vitro study hot acid etching pre-treatment improves the retention of resin cement to zirconia crowns. The use of MDP self-adhesive resin cement (Panavia SA Cement Plus) with hot acid etching is effective and can be used for adhesive cementation of zirconia crowns.

## Figures and Tables

**Table 1:** Study grouping.

Code	Group
AP	The crowns were air abraded using aluminum oxide particles and cemented by Panavia SA Cement Plus
AL	The crowns were air abraded using aluminum oxide particles and cemented by GC LinkForce Cement
AHP	The crowns were air abraded using aluminum oxide particles, etched using the hot acid, and cemented by Panavia SA
AHP	The crowns were air abraded using aluminum oxide particles, etched with the hot acid, and cemented by Panavia SA

**Table 2:** Means and standard deviations of the retention force (N), total surface area (mm^2^), and the retention strength (MPa) for the study groups.

Group	Retention Force (N)	Surface Area (mm^2^)	Retention Strength (Mpa)
AHP	422.54 ± 88.71	109.85 ± 9.24	3.83 ± 0.71
AHL	233.03 ± 54.28	114.54 ± 11.48	2.00 ± 0.52
AP	354.95 ± 30.96	111.24 ± 9.77	3.22 ± 0.35
AL	163.02 ± 31.57	112.36 ± 6.25	1.44 ± 0.22

**Table 3:** Post Hoc test between the mean retention strength of study groups.

Group		Mean Difference (I-J)	Std. Error Sig.	Sig.
AHP	AHL	1.829	0.218	0.000
	AP	0.608	0.218	0.008
	AL	2.39	0.218	0.000
AHL	AP	−1.221	0.218	0.000
	AL	0.562	0.218	0.014
AP	AL	1.783	0.218	0.000

**Table 4:** Two-way ANOVA test regarding the hot acid etching and cement type on the retention strength of zirconia crown.

Variable	Sum of Squares	df	Mean	F	*p*
Etching	3.416	1	3.416	14.439	0.001
Cement	32.5981	1	1	137.769	0.000
Etching × Cement	0.005	1	32.598	0.022	0.882
Error	8.518	36	0.005	-	-
Total	319.911	40	0.237	-	-

**Table 5:** Failure mode of all tested groups.

Group	Adhesive	Cohesive	Mixed
AHP	2	2	6
AHL	5	2	3
AP	3	1	6
AL	10	0	0

## Data Availability

The data presented in this study are available on request from the corresponding author A.S.
